# Etiology and Treatment of Endometritis*Read at Austin District Medical Society, March 23, 1899.

**Published:** 1899-05

**Authors:** J. S. Watson

**Affiliations:** Manor, Texas


For Texas Medical Journal.

### Etiology and Treatment of Endometritis.*

BY J. S. WATSON, M. D., MANOR, TEXAS.

From the earliest ages of our science, many of the best men in the ranks of medicine have given their time and talent to the study of this disease, and to the world the results of their experience and observations; hence, there is a voluminous literature of the subject. Each generation seems to have collected many of the truths of their predecessors and disproven some theories which were erroneous, adding a truth of their own now and then, as well as their theories; thus giving us our modern system of gynecology, of which we are justly proud. You have these works in your libraries, so I shall content myself with giving you the experience of a country doctor in his practical work in this line, with such suggestions from his literature as he has deemed most helpful.

We will first notice a few points in the anatomy of the uterus and adnexa: (1) The uterus in health lies in the superior strait of the pelvis, with its long axis parallel with the axis of the strait. (2) Its nerve supply comes from the first and second sacral, and some branches of the sympathetic which join them in the sacral plexus. (3) Its blood supply comes to the fundus, through the ovarian artery, a branch of the aorta, and to the lower half of the body, and carries through the uterine artery a branch of the internal illiac.

The ovarian artery passes along the upper border of the broad ligament, supplying the ovary and the fundus uteri. The uterine artery passes along the lower margin of the broad ligament, sending some branches to the vagina and cervix; but the main trunk enters the walls of the uterus opposite the internal os, and winds its way upward anastomosing with the ovarian. These, I believe, are the most tortuous vessels in the whole body. They also have their accompanying veins, which, in the walls of the organ, are called "sinuses." (4) The uterus is lined by a mucous membrane which extends through the tubes above, and is continuous with the vaginal mucous membrane below. This membrane, in the endometrium, is supplied with columnar ciliated epithelium. This membrane may be completely thrown off; but beneath it, and embedded in the walls of the organ, we find a series of glands which have the power

---

Read at Austin District Medical Society, March 23, 1899.

of rapidly reforming it, and in the course of four or five weeks it is perfect again.

Etiology: I think by far the best work in modern gynecology is attained by studying such diseases as are always found to exist in the same organ at the same time, coming from the same cause, and which require about the same treatment, as one and the same disease.

This has led some of our best men of the present day to lay aside such distinctions as "cervical" and "corporeal" metritis and endometritis, and treat them all under the more practical name "metritis." I have never seen a case of cervical or corporeal endometritis without some change in the walls of the organ, and our microscopists tell us that this changed interstitial tissue carries the germs of inflammation as well as the mucosa membrane. If I have ever seen a case in which the uterine walls were not involved, it was in the early stages of gonorrheal endometritis, and that remained so limited but a very short time.

Acute metritis may be caused by sepsis, as in the puerperal state; by specific poison, as in the gonorrheal form; or from any other cause which interferes with the nerve or blood supply of the part, as in some fevers,—the acute exanthematous, etc.

This is aptly illustrated in dengue. You have doubtless observed the hypogastric tenderness, the sacral pain, and the intense cephalalgia in these cases, which accompany an acute metritis, and have doubtless also seen this confirmed by the profuse menstrual flow so frequent in these cases, and which seems to have no regard for the time of normal menstruation.

Women sometimes attribute metritis to cold, or to sudden changes, and indeed I see no reason why we should not have an acute metritis from any cause which will produce a mucous catarrh elsewhere, provided the poison has the same chance to reach the organ or its mucous membrane. I have never seen any of these cases become chronic, except the septic and the specific forms. There is, however, another form which comes from stenosis, flexions, displacements, etc., which seem to be chronic, with acute exacerbations; also, traumatism may cause an acute metritis, which, as a rule, passes into the chronic form before it is controlled, and oftentimes before it is discovered.

Stenosis and flexion cause pain, and pain causes congestion, and frequent or continued congestions give rise to hyperplasia; and we have what some of our authors call "interstitial metritis," so also the displacement, by possibly narrowing the ovarian artery and its veins, will interfere somewhat with the circulation in the fundus; but the displacements almost always lower the uterus, thus rendering the already tortuous uterine artery and its veins still more tortuous, impeding the blood current, which, in turn, causes hyperplasia. Laceration of the cervix may be sufficient to cause a metritis, either acute or chronic; but when they are sufficient to cause the chronic form, they also cause displacement to such an extent that I am not sure but what the displacement is the immediate, and the laceration the remote, cause of the trouble.

Most of you have, in the use of the curette, noticed more sponginess in the mucous membrane of the lower part of the body of the uterus, and in the cervix, than in the fundus. This may come from the more frequent injuries of these parts, (and especially of the cervix), in labor, but I believe that the uterine artery and its veins being made more tortuous by displacements, suffer more from this than does the ovarian by its narrowing; therefore there is more mischief in the field of this vessel. We shall notice this again in the treatment.

What we find in practice in acute metritis is: Tenderness over the hypogastrium, back ache in the region of the sacrum, an excess in the mucous flow, or derangements of the menstrual function; sometimes headaches, which may be very intense, and frequently, a little rise of temperature. Digital examination reveals tenderness in the uterus, and sometimes in the parametrium. The organ is somewhat engorged, and a little heavier than normal. We may also note the flexions, lacerations, displacements, etc., by the touch in bimanual palpation.

The speculum is used in the chronic forms, and may reveal abrasions, ulcerations, or lacerations of the cervix. The sound shows cavity of the organ open, instead of having its walls in apposition, as in health. It also shows the walls of the organ are more flaccid than normal, and any grade of change in the mucosa from slight excess of granular tissue, to the more pronounced mucous polypi.

The organ is generally a little deeper than normal, and unless bound by old adhesions it is more movable than in health; thus showing that even the uterine supports are to some extent injured by the disease. The speculum may also show stenosis, diseased cervical glands, or a wrong direction of the uterine axis.

There may be many reflex symptoms, as nausea, headache, palpatation, cold spots on the skin, and, sometimes, localized perspiration. "Globus hystericus," and, indeed, almost every grade of nervous phenomena, from pain in the top of the head, to the severest convulsions, may occur.

Prognosis, favorable, provided the cause can be reached and removed; but not otherwise.

Treatment: The acute form, unless from sepsis or traumatism, or from a specific cause, requires very little treatment. Some sedative, anodyne or antispasmodic, with the judicious use of hot applications, being all that is needed, except rest. I have found great relief in these cases from free enemata of hot water left in the bowel. It makes a splendid hot-water bag, and has the advantage of retaining the heat longer, and placing it nearer the pain, than any other way that I have found. If the first enema comes away, use it again within fifteen minutes, and a little effort will retain the second, and this will modify the pain for ten or twelve hours, and if continued a day or two will dispel the soreness. This is also of great advantage for a day or two in the acute exacerbations of the chronic disease; but should not be continued too long in chronic cases, for fear of producing the rectal atony so often observed in those who have been the most enthusiastic users of enemata.

When the disease becomes chronic, we have a much more serious task on our hands, and indeed one which at times has put us all to our wit's end; but while this is true, a great deal has been done, and with fair success, for the relief of that unfortunate class of women who habitually suffer from chronic metritis.

The treatment should be both constitutional and local; and I might also add, mental and moral. We should do all in our power to build up the broken down health and strength, by the judicious use of wholesome food, and an abundance of fresh air and sunshine, with such exercise as the patient can bear; and if she is not able to exercise herself, we will find great benefit from massage, which should be general.

I have never seen benefit from the so-called "uterine massage," nor can I conceive of any more than temporarily lifting the organ, which is nearly always too low, and the possible improvement in the circulation caused by helping to empty the veins; and this is accomplished much more easily and surely by vaginal air pressure in the genu-pectoral position. General massage, however (as the patient will often express it), seems to give new life, and if continued, will improve the appetite and the normal secretions.

In the food line, the patient should have that which is easily digested, and that which will carry sufficient nutriment to supply the demands of the worn out tissues, without too much bulk, and when the digestion is very bad, as we often find in these cases, we should adopt the reconstructives, supplied in an easy assimilable form by the aid of the apothecary's art; but after all, why use these reconstructives, or why trouble ourselves even about the food, if we neglect to look after the air the patient breathes, and see that it is wholesome and that it is fresh; thus insuring a full portion of oxygen? Peptones, and the so-called reconstructives, even though taken into the blood, are worthless, unless they can be properly used by oxidation when they have reached their destination.

The patient should also have iron; but why use iron, without sunshine? for by this the iron becomes, as it were, vitalized. Four times out of five, the fat person is one who lives indoors; and why? It simply means faulty assimilation. I have seen this in a few cases of chronic metritis, where the patient was confined to her room, and in one remarkable case in which she was bedridden for months, and gained flesh to such an extent that she was not able to be up after the metritis was under control; but by the use of iron, sun baths (with the skin bare), and the free use of massage, she was soon relieved.

As to medicines, strictly; many have been suggested, but, alas, the next generation has in most cases discarded the suggestions of their predecessors. Iron, phosphorus, strychnia, and arsenic, have been my main reliance as tonics, and these may be used in combination or alternately, as the individual case seems to demand. Those used for specific action are ergot, Indian hemp, belladonna, black haw, cimicifuga, etc., together with some of the mineral waters, as Bedford Springs, Va. Ergot is theoretically the proper thing, as it is supposed to cause contraction of the pelvic arteries, which is very much to be desired; but the veins have no such power of muscular contraction, and hence do not share in the benefit; therefore ergot has not given the satisfaction in these cases that it has in some fibroids. Cannabis Indica, cimicifuga, belladonna, black haw, etc., are nervous sedatives, and are useful as pain demands. Bedford Spring water carries iron and alum, and hence is not only a tonic, but an astringent, and may be of much benefit if within our reach. I have found in ammoniated ferric alum an admirable substitute, and have found much benefit from it in cases of menorrhagia, where ergot had failed. I prescribe it in solution so that a fluid dram carries from three to five grains, and of this solution I give one dram in a half glass of hot water three or four times a day. The flow will be much less by the second period, but the treatment should be continued for three or four months.

Local Treatment: The hot vaginal douche comes first, and is useful from an hygienic standpoint, because it keeps the parts clean, and hence more comfortable to the patient. And medicinally, as Emmett has shown, by causing contraction of all the pelvic blood vessels with which it comes in contact, and this treatment being continued for a month or two causes the relaxed vessels to regain their tone, thus helping the cure. Where there is ulceration of the cervix, or when the mucous membrane is tumefied or granulated, we may have recourse to astringents, such as iodine, either alone or combined with carbolic acid, tr. iron, etc.

I have seen a granulated mucous membrane come away from almost the whole of the endometrium, from a cotton swab carrying tr. iron being carried into the cavity of the organ and allowed to remain from two to five minutes, and this gives but little pain. Iodine, carbolic acid, etc., are used the same way, but their effects are not quite so decided.

I need not refer to the stronger caustics, as they are a thing of the past in the treatment of metritis.

In many cases the curette is a valuable aid in treatment, and I think the sharp instrument the most preferable, as it does its work with so much less force, and of course this is best used in experienced hands, but I have never seen harm come from it in the hands of an inexperienced physician. The average country doctor is most likely to err on the other side, for unless the whole of the diseased membrane is taken away, the curettment has failed of its purpose; hence I believe that the application of tincture of iron will do all that the curette will do, and that it will not take much longer, and is, therefore, the safest and surest treatment for the general practitioner.

There are also some "uterine wafers," "tablets,"etc., which carry astringents that are very useful helps when used in the vagina, but they should always be placed behind the cervix, and after remaining a few hours should be washed away with the vaginal douche.

It would seem at first thought that a local application which fails to reach the endometrium could not accomplish good; but like the hot douche, they contract almost every fibre of the parts, and hence have a salutary effect on the uterine artery and its branches with which they come in close touch, as it passes along the lower border of the broad ligament. These also are the vessels most affected by the hot douche, and indeed, they are the vessels that suffer most from the disease.

A tampon of glycerine, or tannate of glycerine, is also valuable. If the case is of specific origin, this should have appropriate treatment with some antiseptic and astringent application or wash, and here again tincture of iron, iodine carbolic acid, etc., are useful. Mercury bi-chloride is not well borne by the endometrium, and hence the solution should be very weak if used at all.

In the septic form, the free use of sterilized water carrying ten to twenty drops of carbolic acid to the gallon, used with the intra-uterine irrigator, has given me the most satisfaction. If the trouble has been caused by cervical or perineal lacerations, they should receive attention as soon as the inflammation is under control; so also displacements, flexions, etc., should receive attention, as they are almost sure to cause a relapse if neglected, as indeed they have, in a great measure, been the cause of the present trouble. Those patients are almost always despondent, and the world goes wrong with them, and a gloom hangs over their homes which betokens ill to themselves or their families, and this very gloom becomes a great obstacle to recovery. These are the cases that will tax our wits most strongly. The family and nurses should be shown these things, and be on their guard to avoid them as much as possible, and to give the patient all the diversion at their command, and if there is a bit of cheery news in the community, the doctor should be sure to stow it away for his next visit. The physician who calls to see these cases and fails to bring a "little sunshine" with him, may almost as well have left off the call. This, however, must always be done in such a way as to bring the much needed diversion, and thus relieve, as much as possible, the mental condition of the patient, but we must also be on our guard to maintain her confidence as her physician, and thus summon her moral being to our assistance.

The old familiar and easy dodge of calling all these cases "hysterics," has done great harm in this line. It has caused the friends to think the sufferer was a malinquerer, and the patient herself to lose confidence in her doctor, and eke out a most miserable existence, when the proper course would have restored her to reasonable health and happiness, and made her a useful member of the family and the community.

I am indebted to Thomas and Emmett, Mason and Pozzi, and their contributions, as my authors.

REMARKS: If I may be allowed a written word or two after the discussion, I would notice Dr. Mathews' two forms of endometritis. One from subinvolution, and one interstitial. This bears out the idea that the body is affected as well as the mucous membrane, and that they should be treated together. This is also true, as the doctor stated, with lacerations. The doctor also feared the curette in inexperienced hands, but as stated before, my greatest fear is that the inexperienced may not be thorough enough in his work.

Dr. Denton prefers the dull instrument, but there are some cases that will not yield to the dull instrument without more pressure than one unused to such work is willing to exert. I had a case of abortion last fall where a bit of the placenta had been left, and it was so firmly adherent that I was afraid to use sufficient force with the dull instrument, but the use of an astringent (possibly tr. iron, I do not remember whether I used the iron or iodine), was followed by cessation of the hemorrhage and the expulsion of the offending mass in about three days.

Dr. Thomas' suggestion of using fifteen drops tr. iron injected into the uterus with an appropriate instrument, is good, and I think destined to replace curettement in many cases.

